# Supplementary material for: Two HEPN domains dictate CRISPR RNA maturation and target cleavage in Cas13d
Source: Nat Commun. 2019 Jun 11;10:2544. doi: 10.1038/s41467-019-10507-3 (PMC6559982; doi:10.1038/s41467-019-10507-3)
Supplement: Supplementary file 1 — Supplementary Information [file 41467_2019_10507_MOESM1_ESM.pdf]

SUPPLEMENTARY INFORMATION

Two HEPN domains dictate CRISPR RNA maturation and target cleavage in Cas13d

B. Zhang, Y. Ye et al.

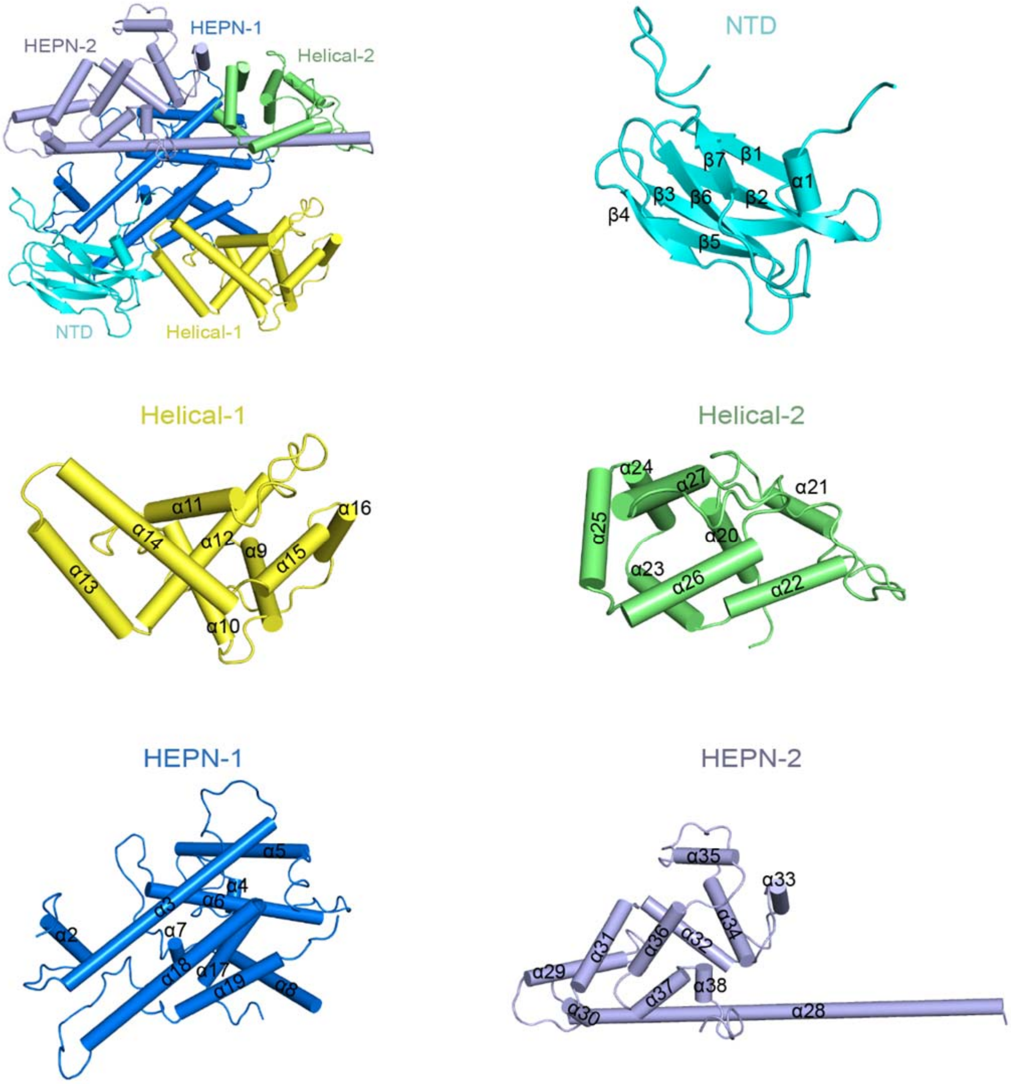

Supplementary Fig. 1 | Structures of individual domains of UrCas13d in the UrCas13d-crRNA binary complex.

Domains are colored according to Fig. 1.

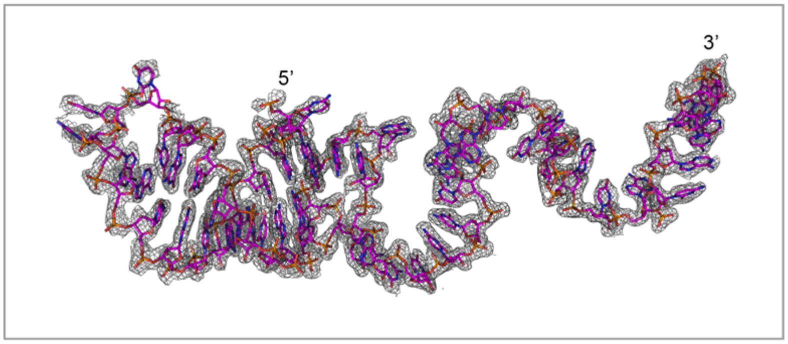

Supplementary Fig. 2 | Electron density maps of crRNAs in the UrCas13d binary complexes.

The electron density map of crRNA in the UrCas13d-crRNA binary complex. A 50-nt crRNA was determined in the binary complex. The 2Fo-Fc omit map was contoured at 1.0 σ level.

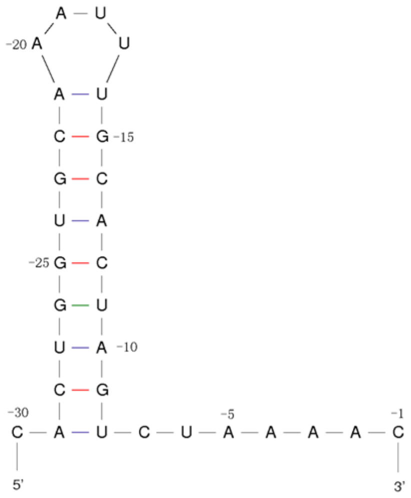

Supplementary Fig. 3 | Predicted secondary structure of the mature crRNA repeat region in the UrCas13d-crRNA binary complex.

Result predicted by the mfold web server (http://unafold.rna.albany.edu/?q=mfold).

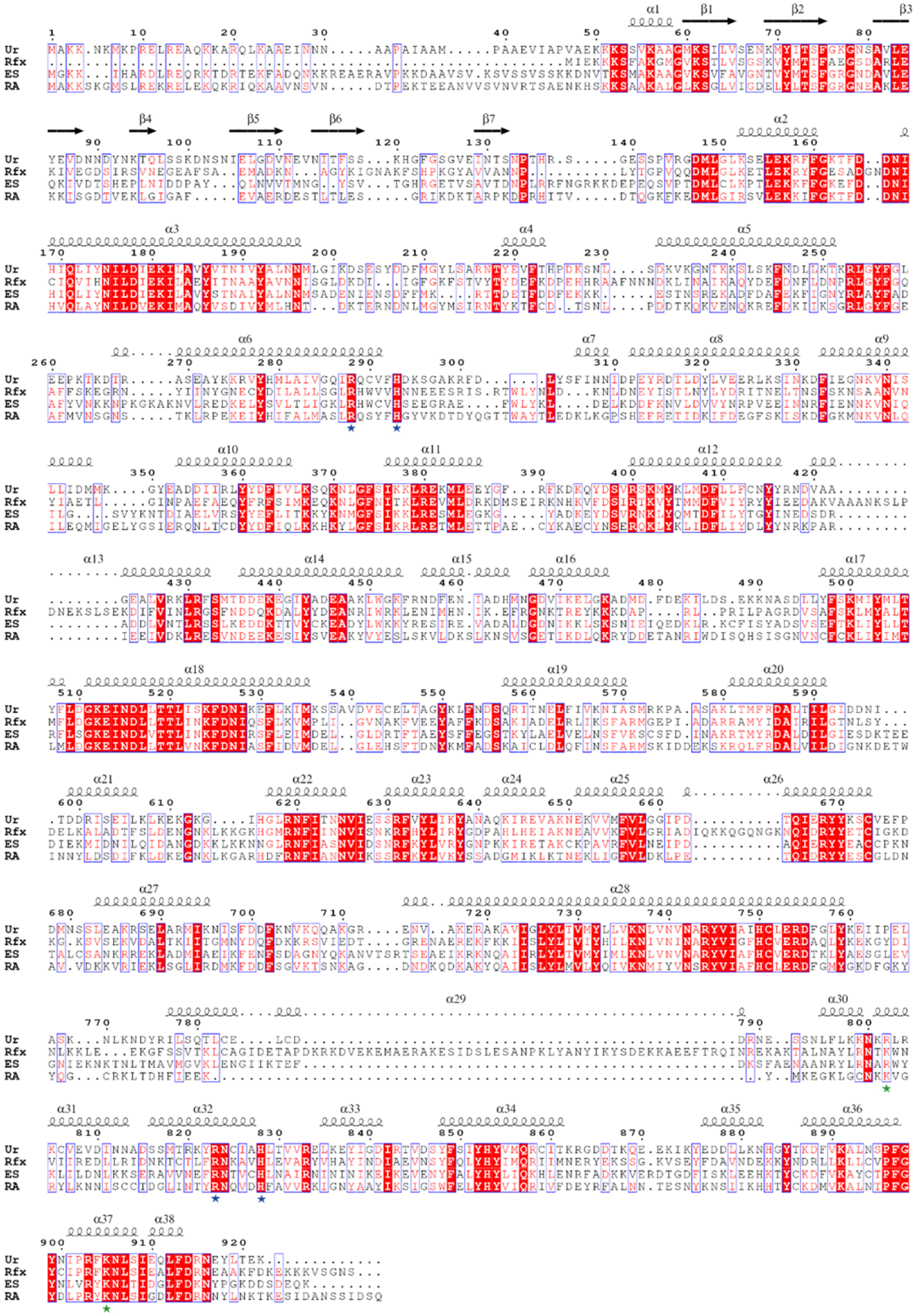
Ur
Rfx
ES
RA

Supplementary Fig. 4 | Multiple sequence alignment of Cas13d proteins from different species.

Sequence alignment of UrCas13d (Uncultured *Ruminococcus sp.* Cas13d), RfxCas13d (*Ruminococcus flavefaciens* XPD3002 Cas13d), EsCas13d (*Eubacterium siraeum* Cas13d) and RaCas13d (*Ruminococcus albus* Cas13d) was generated using Clustal Omega and the figure was prepared using ESPript (http://espript.ibcp.fr). The secondary structure of UrCas13d is shown above the sequence. Identical and similar residues are highlighted in red and white boxes, respectively. Critical residues involved in the pre-crRNA cleavage are marked with green stars, and catalytic residues involved in the target RNA cleavage are marked with blue stars.

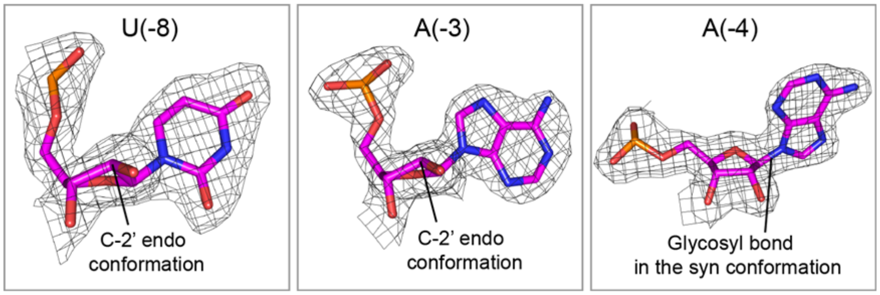

Supplementary Fig. 5 | Conformations of nucleotides U(-8), A(-3) and A(-4) within the U-shaped turn of the crRNA repeat region.

Compared to a standard A-form RNA, nucleotides U(-8), A(-4) and A(-3) within the U-shaped turn have unusual conformations with respect to their sugar pucker or glycosyl bond. The 2Fo-Fc omit map was contoured at 1.0 σ level.

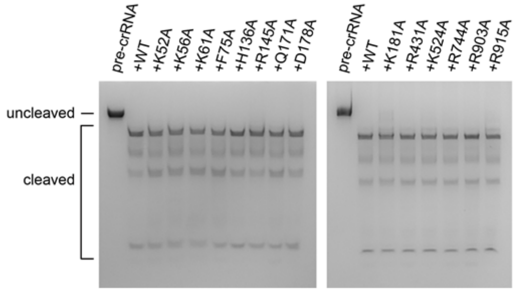

Supplementary Fig. 6 | Pre-crRNA processing by wild-type UrCas13d and its mutants. Denaturing gel demonstrating the cleavage of pre-crRNA by wild-type UrCas13d and its mutants.

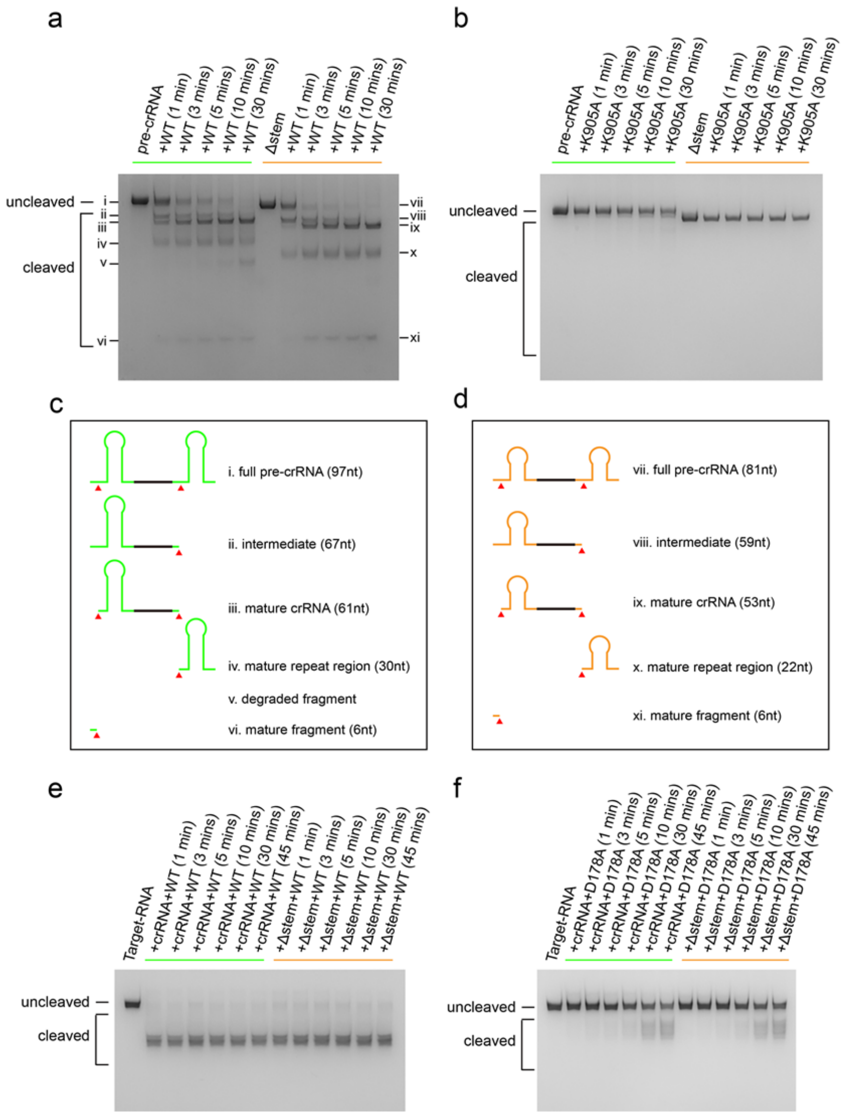

Supplementary Fig. 7 | The time-course cleavage of pre-crRNA and target RNA by UrCas13d and its mutants.

a-b, denaturing gel demonstrating the time-course cleavage of pre-crRNA or the ∆stem mutant by the wild-type UrCas13d or the K905A mutant; **c-d**, the expected sizes of the pre-crRNA cleavage products were labeled as i-vi, and the expected sizes of the ∆stem mutant cleavage products were labeled as vii-xi; **e-f**, denaturing gel demonstrating the time-course cleavage of target RNA by the wild-type UrCas13d or the D178A mutant in the presence of crRNA or the ∆stem mutant. The rationale behind selecting these mutants in the

experiments is that the K905A mutant abolishes the pre-crRNA processing, while the D178A mutant greatly decreases the target RNA cleavage.

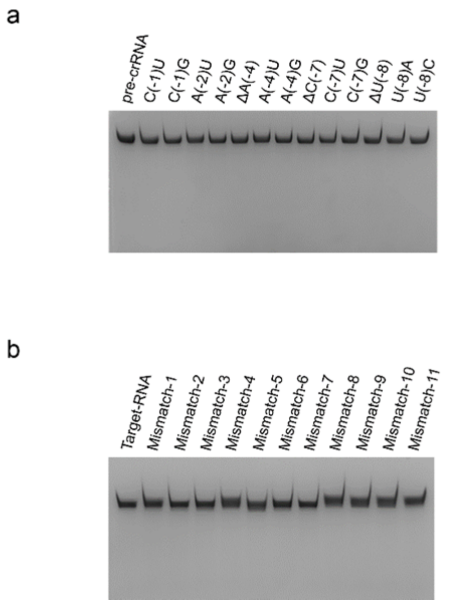

Supplementary Fig. 8 | Pre-crRNA, target RNA and their mutants.

a, Denaturing gel demonstrating pre-crRNA and its mutants. **b**, Denaturing gel demonstrating target RNA and the mismatched mutants.

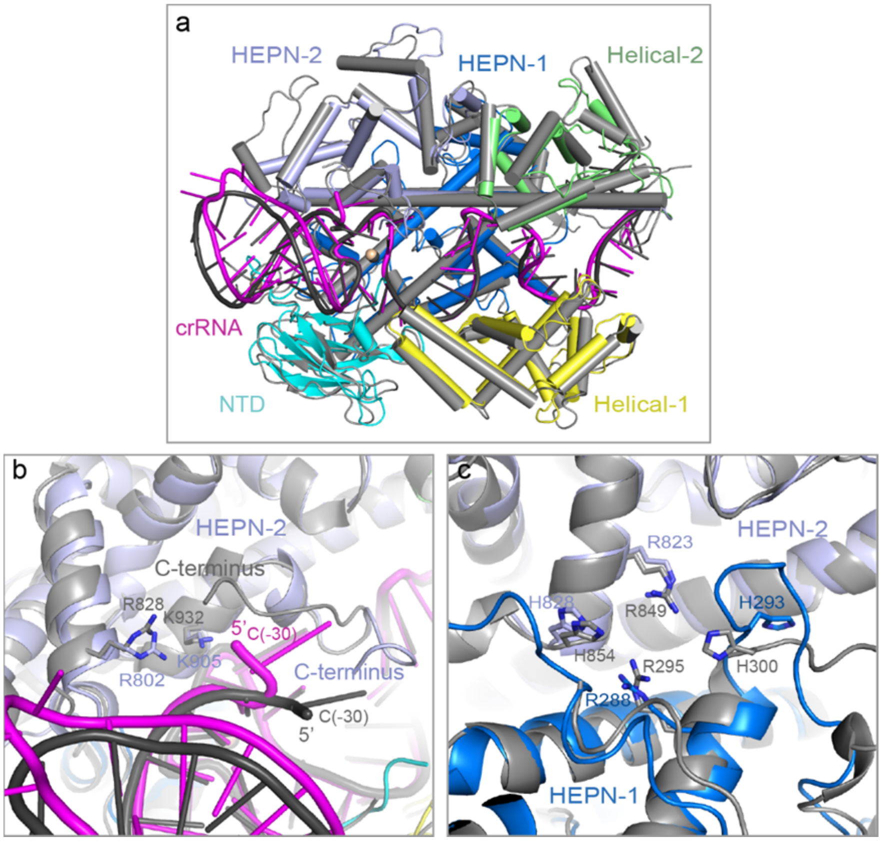

Supplementary Fig. 9 | Comparison of the UrCas13d and EsCas13d binary complexes.

a, Superimposition of the UrCas13d binary complex (PDB code: 6IV9) and the EsCas13d binary complex (PDB code: 6E9E). Domains and crRNA of the UrCas13d binary complex are colored and labeled according to Fig. 1. Domains and crRNA of the EsCas13d complex are colored in gray and black, respectively. **b**, Comparison of the pre-crRNA processing sites in two binary complexes. Two critical residues from HEPN-2 domain of the respective binary complexes are colored and labeled according to (**a**). **c**, Comparison of the target RNA-cleavage sites in two binary complexes. Four catalytic residues from HEPN-1 and HEPN-2 domains of the respective binary complexes are colored and labeled according to (**a**).

Supplementary Table 1 | X-ray crystallography data collection and refinement statistics.

**Dataset**	SeMet-UrCas13d-R288A/R823A-crRNA	UrCas13d-R288A/R823A-crRNA
**Data collection**		
Beamline	BL-17U1, SSRF	BL-17U1, SSRF
Wavelength (Å)	0.9792	0.9792
Space group	$P2_12_12_1$	$P2_12_12_1$
Cell dimensions		
a, b, c (Å)	65.16, 145.73, 249.38	72.22, 136.48, 147.28
α, β, γ (°)	90.0, 90.0, 90.0	90.0, 90.0, 90.0
Resolution range (Å)*	62.91-2.15 (2.19-2.15)	100.11-1.86 (1.89-1.86)
Completeness (%)	99.70 (100.0)	98.90 (98.80)
$I/\sigma(I)$	18.1 (6.3)	21.0 (3.6)
R_{merge}	0.129 (0.842)	0.071 (0.882)
Multiplicity	13.3 (13.9)	13.5 (14.1)
**Refinement**		
Resolution (Å)	2.15	1.86
No. unique reflections	129,626	121,127
R_{work}/ R_{free} (%)	18.9/ 21.0	17.0/ 19.6
No. atoms		
Protein	14,076	7,126
Nucleic acid	2,196	1,054
Solvent	665	475
Mg^{2+}	2	2
B-factors (Å^2)		
Protein	48.36	38.91
Nucleic acid	60.94	42.76
Solvent	37.16	40.55
Mg^{2+}	26.88	44.18
R.m.s deviations		
Bond length (Å)	0.013	0.015
Bond angles (°)	1.78	1.74
Ramachandran plot (%)		
Favored region	98.37	98.39
Allowed region	1.63	1.61
Outliers region	0	0

*Highest resolution shell is shown in parentheses.

Supplementary Table 2 | DNA coding sequence of UrCas13d used in this study.

Description	DNA coding sequence*
UrCas13d	ATGGCAAAAAAAAATAAAATGAAACCGCGTGAACTGCGTGAAGCACAGAAAAAAGC<br>ACGTCAGCTGAAAGCAGCAGAAATTAATAATAATGCAGCACCGGCAATTGCAGCAAT<br>GCCGGCAGCAGAAGTTATTGCACCGGTTGCAGAAAAAAAAAAAAAGCAGCGTTAAAGC<br>AGCAGGTATGAAAAGCATTCTGGTTAGCGAAAATAAAATGTATATTACCAGCTTTGGT<br>AAAGGTAATAGCGCAGTTCTGGAATATGAAGTTGATAATAATGATTATAATAAAACC<br>CAGCTGAGCAGCAAAGATAATAGCAATATTGAACTGGGTGATGTTAATGAAGTTAAT<br>ATTACCTTTAGCAGCAAACATGGTTTTGGTAGCGGTGTTGAAATTAATACCAGCAATC<br>CGACCCATCGTAGCGGTGAAAGCAGCCCGGTTCGTGGTGATATGCTGGGTCTGAAAA<br>GCGAACTGGAAAAACGTTTTTTTGGTAAAACCTTTGATGATAATATTCATATTCAGCT<br>GATTTATAATATTCTGGATATTGAAAAAATTCTGGCAGTTTATGTTACCAATATTGTTT<br>ATGCACTGAATAATATGCTGGGTATTAAAGATAGCGAAAGCTATGATGATTTTATGGG<br>TTATCTGAGCGCACGTAATACCTATGAAGTTTTTACCCATCCGGATAAAAGCAATCTG<br>AGCGATAAAGTTAAAGGTAATATTAAAAAAAGCCTGAGCAAATTTAATGATCTGCTG<br>AAAACCAAACGTCTGGGTTATTTTGGTCTGGAAGAACCGAAAACCAAAGATACCCGT<br>GCAAGCGAAGCATATAAAAAACGTGTTTATCACATGCTGGCAATTGTTGGTCAGATTC<br>GTCAGTGTGTTTTTCATGATAAAAGCGGTGCAAAACGTTTTGATCTGTATAGCTTTATT<br>AATAATATTGATCCGGAATATCGTGATACCCTGGATTATCTGGTTGAAGAACGTCTGA<br>AAAGCATTAATAAAGATTTTATTGAAGGTAATAAAGTTAATATTAGCCTGCTGATTGA<br>TATGATGAAAGGTTATGAAGCAGATGATATTATTCGTCTGTATTATGATTTTATTGTTC<br>TGAAAAGCCAGAAAAATCTGGGTTTTAGCATTAAAAAACTGCGTGAAAAAATGCTGG<br>AAGAATATGGTTTTCGTTTTAAAGATAAACAGTATGATAGCGTTCGTAGCAAAATGTA<br>TAAACTGATGGATTTTCTGCTGTTTTGTAATTATTATCGTAATGATGTTGCAGCAGGTG<br>AAGCACTGGTTCGTAAACTGCGTTTTAGCATGACCGATGATGAAAAAGAAGGTATTTA<br>TGCAGATGAAGCAGCAAAACTGTGGGGTAAATTTCGTAATGATTTTGAAAATATTGCA<br>GATCACATGAATGGTGATGTTATTAAAGAACTGGGTAAAGCAGATATGGATTTTGATG<br>AAAAAATTCTGGATAGCGAAAAAAAAAAATGCAAGCGATCTGCTGTATTTTAGCAAAA<br>TGATTTATATGCTGACCTATTTTCTGGATGGTAAAGAAATTAATGATCTGCTGACCACC<br>CTGATTAGCAAATTTGATAATATTAAAGAATTTCTGAAAATTATGAAAAGCAGCGCAG<br>TTGATGTTGAATGTGAACTGACCGCAGGTTATAAACTGTTTAATGATAGCCAGCGTAT<br>TACCAATGAACTGTTTATTGTTAAAAATATTGCAAGCATGCGTAAACCGGCAGCAAGC<br>GCAAAACTGACCATGTTTCGTGATGCACTGACCATTCTGGGTATTGATGATAATATTA<br>CCGATGATCGTATTAGCGAAATTCTGAAACTGAAAGAAAAAGGTAAAGGTATTCATG<br>GTCTGCGTAATTTTATTACCAATAATGTTATTGAAAGCAGCCGTTTTGTTTATCTGATT<br>AAATATGCAAATGCACAGAAAATTCGTGAAGTTGCAAAAAATGAAAAAGTTGTTATG<br>TTTGTTCTGGGTGGTATTCCGGATACCCAGATTGAACGTTATTATAAAAGCTGTGTTGA<br>ATTTCCGGATATGAATAGCAGCCTGGAAGCAAAACGTAGCGAACTGGCACGTATGAT<br>TAAAAATATTAGCTTTGATGATTTTAAAAATGTTAAACAGCAGGCAAAAGGTCGTGAA<br>AATGTTGCAAAAGAACGTGCAAAAGCAGTTATTGGTCTGTATCTGACCGTTATGTATC<br>TGCTGGTTAAAAATCTGGTTAATGTTAATGCACGTTATGTTATTGCAATTCATTGTCTG<br>GAACGTGATTTTGGTCTGTATAAAGAAATTATTCCGGAACTGGCAAGCAAAAATCTGA

	AAAATGATTATCGTATTCTGAGCCAGACCCTGTGTGAACTGTGTGATGATCGTAATGA<br>AAGCAGCAATCTGTTTCTGAAAAAAAATAAACGTCTGCGTAAATGTGTTGAAGTTGAT<br>ATTAATAATGCAGATAGCAGCATGACCCGTAAATATCGTAATTGTATTGCACATCTGA<br>CCGTTGTTCGTGAACTGAAAGAATATATTGGTGATATTCGTACCGTTGATAGCTATTTT<br>AGCATTTATCATTATGTTATGCAGCGTTGTATTACCAAACGTGGTGATGATACCAAAC<br>AGGAAGAAAAAATTAAATATGAAGATGATCTGCTGAAAAATCATGGTTATACCAAAG<br>ATTTTGTTAAAGCACTGAATAGCCCGTTTGGTTATAATATTCCGCGTTTTAAAAATCTG<br>AGCATTGAACAGCTGTTTGATCGTAATGAATATCTGACCGAAAAATAA

*Codons have been optimized.

Supplementary Table 3 | RNA coding sequences used in the study.

Description	RNA coding sequences*
CRISPR RNA	CTACTACACTGGTGCAAATTTGCACTAGTCTAAAACTCCTCGATT<br>ACATACACAAAGCAGCCTACTACACTGGTGCAAATTTGCACTAG<br>TCTAAAACTCCTCGATTACATACACAAAGCAGCCTACTACACTG<br>GTGCAAATTTGCACTAGTCTAAAACTCCTCGATTACATACACAA<br>AGCAGCCTACTACACTGGTGCAAATTTGCACTAGTCTAAAACTC<br>CTCGATTACATACACAAAGCAGCCTACTACACTGGTGCAAATTT<br>GCACTAGTCTAAAACTCCTCGATTACATACACAAAGCAGCCTAC<br>TACACTGGTGCAAATTTGCACTAGTCTAAAACTCCTCGATTACA<br>TACACAAAGCAGCCTACTACACTGGTGCAAATTTGCACTAGTCT<br>AAAACTCCTCGATTACATACACAAAGCAGCCTACTACACTGGTG<br>CAAATTTGCACTAGTCTAAAAC
Pre-crRNA	CTACTACACTGGTGCAAATTTGCACTAGTCTAAAACTCCTCGATT<br>ACATACACAAAGCAGCCTACTACACTGGTGCAAATTTGCACTAG<br>TCTAAAAC
The ∆U(-8) pre-crRNA mutant	CTACTACACTGGTGCAAATTTGCACTAGCTAAAACTCCTCGATT<br>ACATACACAAAGCAGCCTACTACACTGGTGCAAATTTGCACTAG<br>CTAAAAC
The U(-8)A pre-crRNA mutant	CTACTACACTGGTGCAAATTTGCACTAGACTAAAACTCCTCGAT<br>TACATACACAAAGCAGCCTACTACACTGGTGCAAATTTGCACTA<br>GACTAAAAC
The U(-8)C pre-crRNA mutant	CTACTACACTGGTGCAAATTTGCACTAGCCTAAAACTCCTCGAT<br>TACATACACAAAGCAGCCTACTACACTGGTGCAAATTTGCACTA<br>GCCTAAAAC
The ∆C(-7) pre-crRNA mutant	CTACTACACTGGTGCAAATTTGCACTAGTTAAAACTCCTCGATT<br>ACATACACAAAGCAGCCTACTACACTGGTGCAAATTTGCACTAG<br>TTAAAAC
The C(-7)U pre-crRNA mutant	CTACTACACTGGTGCAAATTTGCACTAGTTTAAAACTCCTCGATT<br>ACATACACAAAGCAGCCTACTACACTGGTGCAAATTTGCACTAG<br>TTTAAAAC
The C(-7)G pre-crRNA mutant	CTACTACACTGGTGCAAATTTGCACTAGTGTAAAACTCCTCGAT<br>TACATACACAAAGCAGCCTACTACACTGGTGCAAATTTGCACTA<br>GTGTAAAAC
The ∆A(-4) pre-crRNA mutant	CTACTACACTGGTGCAAATTTGCACTAGTCTAAACTCCTCGATTA<br>CATACACAAAGCAGCCTACTACACTGGTGCAAATTTGCACTAGT<br>CTAAAC
The A(-4)U pre-crRNA mutant	CTACTACACTGGTGCAAATTTGCACTAGTCTATAACTCCTCGATT<br>ACATACACAAAGCAGCCTACTACACTGGTGCAAATTTGCACTAG<br>TCTATAAC
The A(-4)G pre-crRNA mutant	CTACTACACTGGTGCAAATTTGCACTAGTCTAGAACTCCTCGATT<br>ACATACACAAAGCAGCCTACTACACTGGTGCAAATTTGCACTAG<br>TCTAGAAC
The A(-2)U pre-crRNA mutant	CTACTACACTGGTGCAAATTTGCACTAGTCTAAATCTCCTCGATT<br>ACATACACAAAGCAGCCTACTACACTGGTGCAAATTTGCACTAG<br>TCTAAATC
The A(-2)G pre-crRNA mutant	CTACTACACTGGTGCAAATTTGCACTAGTCTAAAGCTCCTCGATT<br>ACATACACAAAGCAGCCTACTACACTGGTGCAAATTTGCACTAG<br>TCTAAAGC
The C(-1)U pre-crRNA mutant	CTACTACACTGGTGCAAATTTGCACTAGTCTAAAATTCCTCGATT<br>ACATACACAAAGCAGCCTACTACACTGGTGCAAATTTGCACTAG<br>TCTAAAAT
The C(-1)G pre-crRNA mutant	CTACTACACTGGTGCAAATTTGCACTAGTCTAAAAGTCCTCGAT<br>TACATACACAAAGCAGCCTACTACACTGGTGCAAATTTGCACTA<br>GTCTAAAAG
The ∆stem pre-crRNA mutant	CTACTACACTGGAATTCTAGTCTAAAACTCCTCGATTACATACA<br>CAAAGCAGCCTACTACACTGGAATTCTAGTCTAAAAC
crRNA	CACTGGTGCAAATTTGCACTAGTCTAAAACTCCTCGATTACATA<br>CACAAAGCA
The ∆U(-8) crRNA mutant	CACTGGTGCAAATTTGCACTAGCTAAAACTCCTCGATTACATAC<br>ACAAAGCA
The U(-8)A crRNA mutant	CACTGGTGCAAATTTGCACTAGACTAAAACTCCTCGATTACATA

	CACAAAGCA
The U(-8)C crRNA mutant	CACTGGTGCAAATTTGCACTAGCCTAAAACTCCTCGATTACATACACAAAGCA
The ΔC(-7) crRNA mutant	CACTGGTGCAAATTTGCACTAGTTAAAACTCCTCGATTACATACACAAAGCA
The C(-7)U crRNA mutant	CACTGGTGCAAATTTGCACTAGTTTAAAACTCCTCGATTACATACACAAAGCA
The C(-7)G crRNA mutant	CACTGGTGCAAATTTGCACTAGTGTAAAACTCCTCGATTACATACACAAAGCA
The ΔA(-4) crRNA mutant	CACTGGTGCAAATTTGCACTAGTCTAAACTCCTCGATTACATACACAAAGCA
The A(-4)U crRNA mutant	CACTGGTGCAAATTTGCACTAGTCTATAACTCCTCGATTACATACACAAAGCA
The A(-4)G crRNA mutant	CACTGGTGCAAATTTGCACTAGTCTAGAACTCCTCGATTACATACACAAAGCA
The A(-2)U crRNA mutant	CACTGGTGCAAATTTGCACTAGTCTAAATCTCCTCGATTACATACACAAAGCA
The A(-2)G crRNA mutant	CACTGGTGCAAATTTGCACTAGTCTAAAGCTCCTCGATTACATACACAAAGCA
The C(-1)U crRNA mutant	CACTGGTGCAAATTTGCACTAGTCTAAAATTCCTCGATTACATACACAAAGCA
The C(-1)G crRNA mutant	CACTGGTGCAAATTTGCACTAGTCTAAAAGTCCTCGATTACATACACAAAGCA
The Δstem crRNA mutant	CACTGGAATTCTAGTCTAAAACTCCTCGATTACATACACAAAGCA
Target RNA	TATATAGCTTTGTGTATGTAATCGAGGAACAAGCT
The mismatched Target RNA-1	TATATAGCTTTGTGTATGTAATCGAGCTACAAGCT
The mismatched Target RNA-2	TATATAGCTTTGTGTATGTAATCGTCGAACAAGCT
The mismatched Target RNA-3	TATATAGCTTTGTGTATGTAATGCAGGAACAAGCT
The mismatched Target RNA-4	TATATAGCTTTGTGTATGTATACGAGGAACAAGCT
The mismatched Target RNA-5	TATATAGCTTTGTGTATGATATCGAGGAACAAGCT
The mismatched Target RNA-6	TATATAGCTTTGTGTAACTAATCGAGGAACAAGCT
The mismatched Target RNA-7	TATATAGCTTTGTGATTGTAATCGAGGAACAAGCT
The mismatched Target RNA-8	TATATAGCTTTGACTATGTAATCGAGGAACAAGCT
The mismatched Target RNA-9	TATATAGCTTACTGTATGTAATCGAGGAACAAGCT
The mismatched Target RNA-10	TATATAGCAATGTGTATGTAATCGAGGAACAAGCT
The mismatched Target RNA-11	TATATACGTTTGTGTATGTAATCGAGGAACAAGCT

*The mutational nucleotides are colored in red.

Supplementary Table 4 | Primers used for mutagenesis of UrCas13d.

Description	Primers used for mutagenesis*
UrCas13d_K52A_F	CCGGTTGCAGAAAAAAAAAGCAAGCAGCGTTAAAGCAGCA
UrCas13d_K52A_R	TGCTGCTTTAACGCTGCTTGCTTTTTTTTTCTGCAACCGG
UrCas13d_K56A_F	AAAAAAAAAAGCAGCGTTGCAGCAGCAGGTATGAAAAGC
UrCas13d_K56A_R	GCTTTTCATACCTGCTGCTGCAACGCTGCTTTTTTTTTT
UrCas13d_K61A_F	GTTAAAGCAGCAGGTATGGCAAGCATTCTGGTTAGCGAA
UrCas13d_K61A_R	TTCGCTAACCAGAATGCTTGCCATACCTGCTGCTTTAAC
UrCas13d_F75A_F	AAAATGTATATTACCAGCGCAGGTAAAGGTAATAGCGCAG
UrCas13d_F75A_R	CTGCGCTATTACCTTTACCTGCGCTGGTAATATACATTTT
UrCas13d_H136A_F	AATACCAGCAATCCGACCGCACGTAGCGGTGAAAGCAGC
UrCas13d_H136A_R	GCTGCTTTCACCGCTACGTGCGGTCGGATTGCTGGTATT
UrCas13d_R145A_F	GGTGAAAGCAGCCCGGTTGCAGGTGATATGCTGGGTCT
UrCas13d_R145A_R	AGACCCAGCATATCACCTGCAACCGGGCTGCTTTCACC
UrCas13d_Q171A_F	TGATGATAATATTCATATTGCACTGATTTATAATATTCTG
UrCas13d_Q171A_R	CAGAATATTATAAATCAGTGCAATATGAATATTATCATCA
UrCas13d_D178A_F	CTGATTTATAATATTCTGGCAATTGAAAAAATTCTGGCA
UrCas13d_D178A_R	TGCCAGAATTTTTTCAATTGCCAGAATATTATAAATCAG
UrCas13d_K181A_F	AATATTCTGGATATTGAAGCAATTCTGGCAGTTTATGTTAC
UrCas13d_K181A_R	GTAACATAAACTGCCAGAATTGCTTCAATATCCAGAATATT
UrCas13d_R288A_F	GCAATTGTTGGTCAGATTGCACAGTGTGTTTTTCATGAT
UrCas13d_R288A_R	ATCATGAAAAACACACTGTGCAATCTGACCAACAATTGC
UrCas13d_H293A_F	ATTCGTCAGTGTGTTTTTGCAGATAAAAGCGGTGCAAAAC
UrCas13d_H293A_R	GTTTTGCACCGCTTTTATCTGCAAAAACACACTGACGAAT
UrCas13d_K367A_F	TATGATTTTATTGTTCTGGCAAGCCAGAAAAATCTGGG
UrCas13d_K367A_R	CCCAGATTTTTCTGGCTTGCCAGAACAATAAAATCATA
UrCas13d_K370A_F	ATTGTTCTGAAAAGCCAGGCAAATCTGGGTTTTAGCATTA
UrCas13d_K370A_R	TAATGCTAAAACCCAGATTTGCCTGGCTTTTCAGAACAAT
UrCas13d_K402A_F	GTATGATAGCGTTCGTAGCGCAATGTATAAACTGATGGA
UrCas13d_K402A_R	TCCATCAGTTTATACATTGCGCTACGAACGCTATCATAC
UrCas13d_K405A_F	GTTCGTAGCAAAATGTATGCACTGATGGATTTTCTGCTGT
UrCas13d_K405A_R	ACAGCAGAAAATCCATCAGTGCATACATTTTGCTACGAAC
UrCas13d_R431A_F	GCACTGGTTCGTAAACTGGCATTTAGCATGACCGATGAT
UrCas13d_R431A_R	ATCATCGGTCATGCTAAATGCCAGTTTACGAACCAGTGC
UrCas13d_K439A_F	AGCATGACCGATGATGAAGCAGAAGGTATTTATGCAGATG
UrCas13d_K439A_R	CATCTGCATAAATACCTTCTGCTTCATCATCGGTCATGCT
UrCas13d_Y443A_F	GATGAAAAAGAAGGTATTGCAGCAGATGAAGCAGCAAAA
UrCas13d_Y443A_R	TTTTGCTGCTTCATCTGCTGCAATACCTTCTTTTTCATC
UrCas13d_K512A_F	ACCTATTTTCTGGATGGTGCAGAAATTAATGATCTGCTG
UrCas13d_K512A_R	CAGCAGATCATTAATTTCTGCACCATCCAGAAAATAGGT
UrCas13d_N515A_F	CTGGATGGTAAAGAAATTGCAGATCTGCTGACCACCCTGA
UrCas13d_N515A_R	TCAGGGTGGTCAGCAGATCTGCAATTTCTTTACCATCCAG
UrCas13d_K524A_F	CTGACCACCCTGATTAGCGCATTTGATAATATTAAAGAA
UrCas13d_K524A_R	TTCTTTAATATTATCAAATGCGCTAATCAGGGTGGTCAG
UrCas13d_K567A_F	AATGAACTGTTTATTGTTGCAAATATTGCAAGCATGCG
UrCas13d_K567A_R	CGCATGCTTGCAATATTTGCAACAATAAACAGTTCATT
UrCas13d_R619A_F	AAAGGTATTCATGGTCTGGCAAATTTTATTACCAATAATG
UrCas13d_R619A_R	CATTATTGGTAATAAAATTTGCCAGACCATGAATACCTTT
UrCas13d_N620A_F	GGTATTCATGGTCTGCGTGCATTTATTACCAATAATGTTA
UrCas13d_N620A_R	TAACATTATTGGTAATAAATGCACGCAGACCATGAATACC
UrCas13d_R631A_F	AATGTTATTGAAAGCAGCGCATTTGTTTATCTGATTAAA
UrCas13d_R631A_R	TTTAATCAGATAAACAAATGCGCTGCTTTCAATAACATT
UrCas13d_K717A_F	GGTCGTGAAAATGTTGCAGCAGAACGTGCAAAAGCAGTTA
UrCas13d_K717A_R	TAACTGCTTTTGCACGTTCTGCTGCAACATTTTCACGACC
UrCas13d_K736A_F	GTTATGTATCTGCTGGTTGCAAATCTGGTTAATGTTAAT
UrCas13d_K736A_R	ATTAACATTAACCAGATTTGCAACCAGCAGATACATAAC
UrCas13d_N737A_F	ATGTATCTGCTGGTTAAAGCACTGGTTAATGTTAATGCAC
UrCas13d_N737A_R	GTGCATTAACATTAACCAGTGCTTTAACCAGCAGATACAT
UrCas13d_R744A_F	CTGGTTAATGTTAATGCAGCATATGTTATTGCAATTCAT
UrCas13d_R744A_R	ATGAATTGCAATAACATATGCTGCATTAACATTAACCAG
UrCas13d_R802A_F	GTTTCTGAAAAAAAATAAAGCACTGCGTAAATGTGTTGAA
UrCas13d_R802A_R	TTCAACACATTTACGCAGTGCTTTATTTTTTTTCAGAAAC

UrCas13d_L803A_F	CTGAAAAAAAATAAACGTGCACGTAAATGTGTTGAAGTT
UrCas13d_L803A_R	AACTTCAACACATTTACGTGCACGTTTATTTTTTTTCAG
UrCas13d_C806A_F	AATAAACGTCTGCGTAAAGCAGTTGAAGTTGATATTAATAATG
UrCas13d_C806A_R	CATTATTAATATCAACTTCAACTGCTTTACGCAGACGTTTATT
UrCas13d_R823A_F	AGCATGACCCGTAAATATGCAAATTGTATTGCACATCTG
UrCas13d_R823A_R	CAGATGTGCAATACAATTTGCATATTTACGGGTCATGCT
UrCas13d_H828A_F	ATCGTAATTGTATTGCAGCACTGACCGTTGTTCGTGAAC
UrCas13d_H828A_R	GTTCACGAACAACGGTCAGTGCTGCAATACAATTACGAT
UrCas13d_K887A_F	GAAAAATCATGGTTATACCGCAGATTTTGTTAAAGCACTGA
UrCas13d_K887A_R	TCAGTGCTTTAACAAAATCTGCGGTATAACCATGATTTTTC
UrCas13d_K891A_F	TATACCAAAGATTTTGTTGCAGCACTGAATAGCCCGTTTG
UrCas13d_K891A_R	CAAACGGGCTATTCAGTGCTGCAACAAAATCTTTGGTATA
UrCas13d_R903A_F	GTTTGGTTATAATATTCCGGCATTTAAAAATCTGAGCATT
UrCas13d_R903A_R	AATGCTCAGATTTTTAAATGCCGGAATATTATAACCAAAC
UrCas13d_F904A_F	GGTTATAATATTCCGCGTGCAAAAAATCTGAGCATTGAA
UrCas13d_F904A_R	TTCAATGCTCAGATTTTTTGCACGCGGAATATTATAACC
UrCas13d_K905A_F	TATAATATTCCGCGTTTTGCAAATCTGAGCATTGAACAG
UrCas13d_K905A_R	CTGTTCAATGCTCAGATTTGCAAAACGCGGAATATTATA
UrCas13d_I909A_F	CGTTTTAAAAATCTGAGCGCAGAACAGCTGTTTGATCGT
UrCas13d_I909A_R	ACGATCAAACAGCTGTTCTGCGCTCAGATTTTTAAAACG
UrCas13d_Q911A_F	TAAAAATCTGAGCATTGAAGCACTGTTTGATCGTAATGAAT
UrCas13d_Q911A_R	ATTCATTACGATCAAACAGTGCTTCAATGCTCAGATTTTTA
UrCas13d_R915A_F	CATTGAACAGCTGTTTGATGCAAATGAATATCTGACCGAA
UrCas13d_R915A_R	TTCGGTCAGATATTCATTTGCATCAAACAGCTGTTCAATG

*Forward and reverse primers are indicated by “F” and “R”, respectively.

Supplementary Table 5 | Primers used for mutagenesis of RNA coding sequences.

Description	Primers used for mutagenesis*
The ΔU(-8) crRNA mutant_F	GGTGCAAATTTGCACTAGCTAAAACTCCTCGATTACA
The ΔU(-8) crRNA mutant_R	TGTAATCGAGGAGTTTTAGCTAGTGCAAATTTGCACC
The U(-8)A crRNA mutant_F	GGTGCAAATTTGCACTAGACTAAAACTCCTCGATTACA
The U(-8)A crRNA mutant_R	TGTAATCGAGGAGTTTTAGTCTAGTGCAAATTTGCACC
The U(-8)C crRNA mutant_F	GGTGCAAATTTGCACTAGCCTAAAACTCCTCGATTACA
The U(-8)C crRNA mutant_R	TGTAATCGAGGAGTTTTAGGCTAGTGCAAATTTGCACC
The ΔC(-7) crRNA mutant_F	GTGCAAATTTGCACTAGTTAAAACTCCTCGATTACA
The ΔC(-7) crRNA mutant_R	TGTAATCGAGGAGTTTTAACTAGTGCAAATTTGCAC
The C(-7)U crRNA mutant_F	GTGCAAATTTGCACTAGTTTAAAACTCCTCGATTACA
The C(-7)U crRNA mutant_R	TGTAATCGAGGAGTTTTAAACTAGTGCAAATTTGCAC
The C(-7)G crRNA mutant_F	GTGCAAATTTGCACTAGTGTAAAACTCCTCGATTACA
The C(-7)G crRNA mutant_R	TGTAATCGAGGAGTTTTACACTAGTGCAAATTTGCAC
The ΔA(-4) crRNA mutant_F	GCAAATTTGCACTAGTCTAAACTCCTCGATTACATACA
The ΔA(-4) crRNA mutant_R	TGTATGTAATCGAGGAGTTTAGACTAGTGCAAATTTGC
The A(-4)U crRNA mutant_F	GCAAATTTGCACTAGTCTATAACTCCTCGATTACATACA
The A(-4)U crRNA mutant_R	TGTATGTAATCGAGGAGTTATAGACTAGTGCAAATTTGC
The A(-4)G crRNA mutant_F	GCAAATTTGCACTAGTCTAGAACTCCTCGATTACATACA
The A(-4)G crRNA mutant_R	TGTATGTAATCGAGGAGTTCTAGACTAGTGCAAATTTGC
The A(-2)U crRNA mutant_F	AATTTGCACTAGTCTAAATCTCCTCGATTACATACAC
The A(-2)U crRNA mutant_R	GTGTATGTAATCGAGGAGATTTAGACTAGTGCAAATT
The A(-2)G crRNA mutant_F	AATTTGCACTAGTCTAAAGCTCCTCGATTACATACAC
The A(-2)G crRNA mutant_R	GTGTATGTAATCGAGGAGCTTTAGACTAGTGCAAATT
The C(-1)U crRNA mutant_F	AATTTGCACTAGTCTAAAATTCCTCGATTACATACAC
The C(-1)U crRNA mutant_R	GTGTATGTAATCGAGGAATTTTAGACTAGTGCAAATT
The C(-1)G crRNA mutant_F	AATTTGCACTAGTCTAAAAGTCCTCGATTACATACAC
The C(-1)G crRNA mutant_R	GTGTATGTAATCGAGGACTTTTAGACTAGTGCAAATT
The mismatched Target RNA-1_F	AGCTTTGTGTATGTAATCGAGCTACAAGCTTGGCGTAATCATG
The mismatched Target RNA-1_R	CATGATTACGCCAAGCTTGTAGCTCGATTACATACACAAAGCT
The mismatched Target RNA-2_F	TAGCTTTGTGTATGTAATCGTCGAACAAGCTTGGCGTAATC
The mismatched Target RNA-2_R	GATTACGCCAAGCTTGTTCGACGATTACATACACAAAGCTA
The mismatched Target RNA-3_F	TATAGCTTTGTGTATGTAATGCAGGAACAAGCTTGGCGTAATC
The mismatched Target RNA-3_R	GATTACGCCAAGCTTGTTCCTGCATTACATACACAAAGCTATA
The mismatched Target RNA-4_F	GTATATAGCTTTGTGTATGTATACGAGGAACAAGCTTGGCGTA
The mismatched Target RNA-4_R	TACGCCAAGCTTGTTCCTCGTATACATACACAAAGCTATATAC
The mismatched Target RNA-5_F	GGTATATAGCTTTGTGTATGATATCGAGGAACAAGCTTGGCGT
The mismatched Target RNA-5_R	ACGCCAAGCTTGTTCCTCGATATCATACACAAAGCTATATACC
The mismatched Target RNA-6_F	TAGGTATATAGCTTTGTGTAACTAATCGAGGAACAAGCTTGG
The mismatched Target RNA-6_R	CCAAGCTTGTTCCTCGATTAGTTACACAAAGCTATATACCTA
The mismatched Target RNA-7_F	TATAGGTATATAGCTTTGTGATTGTAATCGAGGAACAAGCTTG
The mismatched Target RNA-7_R	CAAGCTTGTTCCTCGATTACAATCACAAAGCTATATACCTATA
The mismatched Target RNA-8_F	ACTATAGGTATATAGCTTTGACTATGTAATCGAGGAACAAGC
The mismatched Target RNA-8_R	GCTTGTTCCTCGATTACATAGTCAAAGCTATATACCTATAGT
The mismatched Target RNA-9_F	CACTATAGGTATATAGCTTACTGTATGTAATCGAGGAACAA
The mismatched Target RNA-9_R	TTGTTCCTCGATTACATACAGTAAGCTATATACCTATAGTG
The mismatched Target RNA-10_F	ACTCACTATAGGTATATAGCAATGTGTATGTAATCGAGGAAC
The mismatched Target RNA-10_R	GTTCCTCGATTACATACACATTGCTATATACCTATAGTGAGT
The mismatched Target RNA-11_F	CGACTCACTATAGGTATATACGTTTGTGTATGTAATCGAGGA
The mismatched Target RNA-11_R	TCCTCGATTACATACACAAACGTATATACCTATAGTGAGTCG

*Forward and reverse primers are indicated by “F” and “R”, respectively.
