# Supplementary material for: Two HEPN domains dictate CRISPR RNA maturation and target cleavage in Cas13d
Source: Nat Commun. 2019 Jun 11;10:2544. doi: 10.1038/s41467-019-10507-3 (PMC6559982; doi:10.1038/s41467-019-10507-3)
Supplement: Supplementary file 3 — Description of Additional Supplementary Files [file 41467_2019_10507_MOESM3_ESM.pdf]

Descriptions of Additional Supplementary Files

File name: Supplementary Movie 1

Description: Domain architecture, Surface representations and electrostatic potential surface of the UrCas13d binary complex. UrCas13d consists of the N-terminal domain (NTD), two Helical domains (Helical-1 and Helical-2), two HEPN domains (HEPN-1 and HEPN-2). With the exception of NTD, the protein is predominantly α-helical. In the binary complex, crRNA is sandwiched in a positive channel formed by multiple domains of UrCas13d. crRNA consists of a 30-nt repeat region and a 20-nt spacer region. The crRNA repeat region displays stem-loop architecture.

File name: Supplementary Movie 2

Description: Detailed interactions between the penta-hydrated Mg^{2+} ion and its surrounding nucleotides, residues. The penta-hydrated Mg^{2+} ion is located at the center of an U-shaped turn formed by nucleotides U(-8)-A(-2) of the crRNA repeat region. Here, the penta-hydrated Mg^{2+} ion forms multiple interactions both with crRNA and UrCas13d. The magnesium ion directly coordinates with five water molecules and an oxygen atom from the phosphate group of the nucleotide C(-7). In addition, the $(Mg(H_2O)_5)^{2+}$ ion hydrogen bonds with the phosphate groups of the nucleotides A(-5), A(-3) and A(-2), as well as the side chain of the residue D178. Besides D178, the residues K181 and K524 are also involved in stabilizing the $(Mg(H_2O)_5)^{2+}$ ion and the coordinated nucleotides.

File name: Supplementary Movie 3

Description: Detailed interactions between the tetra-hydrated Mg^{2+} ion and its surrounding residues, nucleotides. The tetra-hydrated Mg^{2+} ion aids in maintaining the conformation of a loop region in NTD that interacts with crRNA repeat region. More specifically, the tetra-hydrated Mg^{2+} ion hydrogen bonds with backbone atoms of residues T135, R137, S141 and S142 within the loop region. Concurrently, the side chains of another two residues from this loop region, H136 and S138, point towards crRNA to form hydrogen bonds with the phosphate groups of U(-24) and G(-25), respectively. These interactions aid in conformational stabilization of the crRNA repeat region.

File name: Supplementary Movie 4

Description: The active site for pre-crRNA processing in the UrCas13d binary complex. According to the results of our structural and mutational studies, the active site for pre-crRNA processing is located in HEPN-2 domain, in close proximity to the nucleotide C(-30) of the crRNA repeat region. More particularly, the conserved basic residues R802 and K905 are critical for pre-crRNA processing.

File name: Supplementary Movie 5

Description: The active site for target RNA cleavage in the UrCas13d binary complex. Residues A288 and A823 of the binary complex was virtually mutated to R288 and R823. Target RNA cleavage by UrCas13d is carried out by two adjacent R-X_4-H HEPN ribonuclease motifs. The first motif, located in HEPN-1 domain, comprises catalytic residues R288 and H293, whereas the second motif, located in HEPN-2 domain, comprises catalytic residues R823 and H828.
